# The puzzling clinical presentation of fluoropyrimidines cardiotoxicity

**DOI:** 10.3389/fcvm.2022.960240

**Published:** 2022-09-14

**Authors:** Linda Cucciniello, Ettore Bidoli, Elda Viel, Maria Laura Canale, Lorenzo Gerratana, Chiara Lestuzzi

**Affiliations:** ^1^Department of Oncology, Centro di Riferimento Oncologico, Istituto di Ricovero e Cura a Carattere Scientifico (IRCCS), National Cancer Institute, Aviano, Italy; ^2^Unit of Cancer Epidemiology, Centro di Riferimento Oncologico, Istituto di Ricovero e Cura a Carattere Scientifico (IRCCS), National Cancer Institute, Aviano, Italy; ^3^Department of Cardiology, Azienda Sanitaria Friuli Occidentale, ASFO, Pordenone, Italy; ^4^Ospedale Versilia, Azienda Usl Toscana nord ovest, Lido di Camaiore, Italy; ^5^Department of Medical Oncology, Aviano Oncology Reference Center (IRCCS), Aviano, Italy

**Keywords:** cardiotoxicity, cardiotoxicity after chemotherapy, capecitabine, fluorouracil/adverse effects, fluoropyrimidine chemotherapeutics, fluoropyrimidine cardiotoxicity, 5-fluorouracil, capecitabine cardiotoxicity

## Abstract

The cardiotoxicity of fluoropyrimidines (FP) [5-Fluorouracil and Capecitabine] is often reported as acute cardiac ischemia with rest typical angina, signs of ischemia at electrocardiogram (ECG), and ventricular kinetics abnormalities. However, silent ischemia, effort-related toxicity, and ventricular arrhythmias (VA) have been also described. The aim of this study is to report a consecutive series of 115 patients with FP cardiotoxicity observed in a single center both within clinical prospective studies and during the clinical routine. The clinical presentation widely varied as regards symptoms, ECG abnormalities, and clinical outcomes. We report also the strategies used to prevent cardiotoxicity in a subgroup of 35 patients who continued o rechallenged FP therapy after cardiotoxicity. In nearly half of the patients, the cardiotoxicity was triggered by physical effort. Typical angina was rare: the symptoms were absent in 51% of cases and were atypical in half of the other cases. ST-segment elevation and VA were the most frequent ECG abnormality; however, ST segment depression or negative T waves were the only abnormalities in 1/3 of the cases. Troponins essays were often within the normal limits, even in presence of extensive signs of ischemia. The most effective strategy to prevent cardiotoxicity at rechallenge was reducing FP dosage and avoiding physical effort. Anti-ischemic therapies were not always effective. Raltitrexed was a safe alternative to FP. Fluoropyrimidine cardiotoxicity shows a wide variety of clinical presentations in real life, from silent ischemia to atypical symptoms, acute coronary syndrome, left ventricular dysfunction (LVD), VA, or complete atrio-ventricular block. Physical effort is the trigger of cardiotoxicity in nearly half of the cases. The recognition of cardiotoxicity cannot rely on symptoms only but requires an active screening with ECG and stress test in selected cases.

## Background

The fluoropyrimidines (FP) 5-fluorouracil (5-FU) and its prodrug capecitabine represent a mainstay of chemotherapy (CT) regimens for different types of malignancies, including head/neck, gastrointestinal, liver, and breast cancer. They can both induce cardiac toxicity (TOX), mostly in the form of myocardial ischemia (MI), ventricular arrhythmias (VA), left ventricular dysfunction (LVD), and sudden death (SD) (1–7). TOX of FP can be precipitated by effort and it can be asymptomatic, thus leading to an underdiagnosis in retrospective studies (8, 9). According to the literature, the most frequent clinical presentation of FP cardiotoxicity is angina with ST-segment elevation detected at Electrocardiogram (ECG) and mimicking vasospastic angina (Table 1). However, some prospective studies with Holter monitoring have reported transient asymptomatic ST segment elevation and ECG abnormalities different from ST-segment elevation have also been described (mostly ST segment depression and negative T waves) (10). A recent review analyzing data from 37 papers including the original data, reported wide variability in clinical presentation and risk factors, probably attributable to the different definitions provided for TOX and to the different modalities of data collection (11).

**TABLE 1:** Fluoropyrimidine Cardiotoxity reported in the literature.

Author	Title	Type	N cases	ECG ischemia	Angina	ARRH	AMI	Cardiac arrest/death
Saif et al. (31)	Fluoropyrimidine-associated cardiotoxicity: revisited.	Literature review *1	377	69%	45%	23%	22%	1.4%
Robben et al. (29)	The syndrome of 5-fluorouracil cardiotoxicity. An elusive cardiopathy	Review of case reports *1	135	75%	85%	15%	10%	13%
Dyhl-Polk et al. (20)	Cardiotoxicity in cancer patients treated with 5-fluorouracil or capecitabine: a systematic review of incidence, manifestations and predisposing factors	Review *1	94	6-33%		0-2%		0-2%
Zafar et al. (26)	The Incidence, Risk Factors, and Outcomes With 5-Fluorouracil– Associated Coronary Vasospasm	Retrospective analysis *1	87	73%	96%			
Khan et al. (24)	A retrospective study of cardiotoxicities induced by 5-fluouracil (5-FU) and 5-FU based chemotherapy regimens in Pakistani adult cancer patients at Shaukat Khanum Memorial Cancer Hospital & Research Center	Retrospective study *1	60	30%	37%%	81.6%	0%	3.3%
Dyhl-Polk et al. (27)	Incidence and risk markers of 5-fluorouracil and capecitabine cardiotoxicity in patients with colorectal cancer	Retrospective study *1	103	33%	43.6%		22.3%	9.7%
de Forni et al. (22)	Cardiotoxicity of high-dose continuous infusion fluorouracil: a prospective clinical study	Prospective study *1	28	64%	64%	3.5%		28.5%
Peng et al. (10)	Cardiotoxicity of 5-fluorouracil and capecitabine in Chinese patients: a prospective study	Prospective study *1	161	65.2%		68.3%	3.7%	
Kosmas et al. (3)	Cardiotoxicity of fluoropyrimidines in different schedules of administration: a prospective study	Prospective study *1	26	30%	42.3%	46.1%	30.7%	3.8%
Lestuzzi et al. (9)	Effort myocardial ischemia during chemotherapy with 5-fluorouracil: an underestimated risk	Prospective study *1	37 (21 at rest, 16 under effort)	95%	42%	50%	8.22%	
Dyhl-Polk et al. (29)	Myocardial Ischemia Induced by 5-Fluorouracil: A Prospective Electrocardiographic and Cardiac Biomarker Study	Prospective study *2	108 patients evaluated			1.85%	18.7%	0.92%
Lestuzzi et al. (14)	Cardiotoxicity from Capecitabine Chemotherapy: Prospective Study of Incidence at Rest and During Physical Exercise	Prospective study *1	32	100%	46.8%	53.1%		

*1 Percentages reported in relation to those experiencing a cardiotoxicity event. 2 Percentages reported in the relation to the whole group.

## Aim of the study

To describe the clinical presentation of FP cardiotoxicity in patients treated with FP in a single Institution: CRO, National Cancer Institute of Aviano (PN, Italy) from 2001 to 2021, and to report the possibility of cardioprotection strategies in a group of patients who underwent a rechallenge therapy with FP after the first episode of cardiotoxicity.

## Materials and methods

We searched the electronic database of the Cardiology Unit of the CRO from 2001 to 2021 and identified 141 patients who had been classified as having had FP cardiotoxicity. The clinical cardiologic and oncologic charts were reviewed by expert cardio-oncologists, in order to confirm the diagnosis and to collect data regarding the clinical history (before and after the diagnosis) whenever available. FP cardiotoxicity was defined as the presence of clinical, ECG, and/or echocardiographic signs of ischemia, Lown >2 VA, supraventricular arrhythmias, complete atrio-ventricular block, or LVD.

Symptoms were classified as follows: “typical chest pain” included typical angina (retrosternal constrictive or squeezing chest pain, radiated or not to the left arm or to jaws) and weight over the sternum; “atypical chest pain” included less defined chest pain or discomfort, burning sensation; “atypical symptoms” included jaw pain, throat constriction, malaise, dizziness, dyspnea.

The diagnosis of TOX had to be confirmed by the disappearance of clinical and instrumental abnormalities after withdrawing FP and by the exclusion of other causes of ischemia or arrhythmias.

**FIGURE 1:**
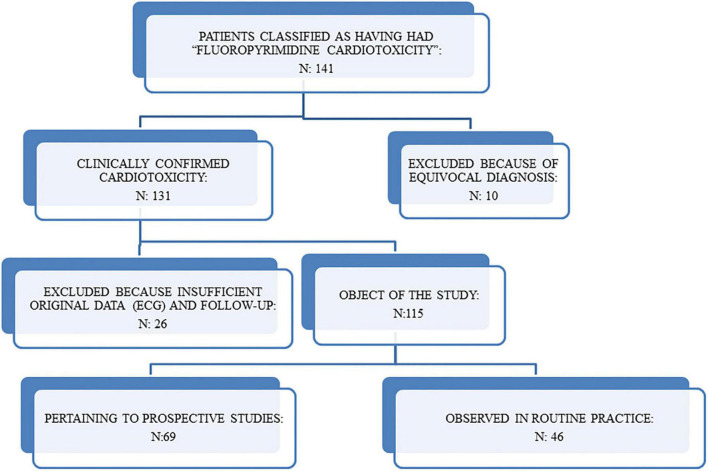
Selection of population reported in the study.

After revision, 10 patients were excluded because the clinical diagnosis of cardiotoxicity was equivocal and another 26 patients (including two patients who died suddenly at home at the end of the 5-FU infusion) were excluded because it was not possible to collect detailed information about the ECG and the cardiovascular risk factors. The remaining 115 pts (74 males and 41 females, aged 19 to 79, mean 59 +11, median 61) are the object of our study (Figure 1). The cases had been observed both in daily practice and in two prospective studies where an effort stress test (EST) was obtained during FP treatment. We investigated also the clinical course of the patients in whom FP, after an episode of cardiotoxicity, was not discontinued or was later re-introduced in the therapy.

## Results

Amongst the 115 patients evaluated in the present study, 79 had at least one CVRF, 15 had a clinical history of ischemic heart disease and 41 were on medical therapy with one or more cardiovascular drugs (in particular, 8 with calcium-channel blockers, 11 with beta-blockers, 22 with angiotensin-converting enzyme inhibitors, and 9 with nitrates). The FP administered was 5-FU in 64 patients and capecitabine in the remaining 51 patients (Table 2).

**TABLE 2:** Characteristics of the patients.

Sex	Males 74
	Females 41
Age	19–79 years (median 61)
Tumor	Liver. 3
	Stomach, gut: 68
	Head, neck: 24
	Breast: 12
	Others: 8
Cardiovascular risk factors (CVRF)	Obesity: 8
	Diabetes: 8
	Hypertension: 41
	Active Smoking: 31
At least 1 CVRF	79
2 or more CVRF	41
Ischemic heart disease	15
On cardiovascular treatment	Beta-blockers: 11
	Calcium channel antagonists: 8
	Angiotensin Converting Enzime inhibitors: 22
	Nitrates: 9
Chemotherapy	5-Fluorouracil: 64
	Capecitabine: 51

Cardiotoxicity (Table 3) was observed at rest in 63 patients and during physical effort in 52 patients. Furthermore, effort-related symptoms during daily life were reported by 10 patients who had cardiotoxicity confirmed by the EST. The ECG recorded at the time of cardiotoxicity diagnosis showed ischemic repolarization changes in 96 patients: ST-segment elevation (1 to 7 mm) in 53 patients, ST segment depression (1 to 7.5 mm) in 16, both ST-segment elevation and depression in 12 patients; negative T waves only in 15 patients. The number of ECG leads showing ST-T changes of ischemia ranged from 2 to 12 (median 5).

**TABLE 3:** Symptoms, ECG and echocardiographic changes at rest and during stress.

ECG changes		N	Within group	Within all 115 patients
At rest (*n* = 62)	ST segment elevation	37	60%	32%
	ST segment depression	4	6%	3%
	Both ST elevation and depression	5	8%	4%
	Negative T waves	13	21%	11%
	Ventricular Ectopic Betas	5	6%	3%
Under/after effort (*n* = 49)	ST segment elevation	15	31%	13%
	ST segment depression	13	27%	11%
	Both ST elevation and depression	7	14%	6%
	Negative T waves	2	4%	2%
	Arrhythmias *	27	55%	23%
**Symptoms**				
**No : 53 (46%)**				

**Yes: 62 (54%)**		**N**	**Within group**	**Within all 115 patients**

Symptoms at rest (*n* = 30)	Typical chest pain	23	77%	20%
	Atypical chest pain	2	7%	2%
	Dyspnoea, dizziness, other atypical symptoms	5	17%	4%
Symptoms under effort (*n* = 32)	Typical chest pain	16	50%	14%
	Atypical chest pain	7	22%	6%
	Dyspnoea, dizziness, other atypical symptoms	9	28%	8%
Echocardiogram (*n* = 35)	Global dysfunction	9	26%	8%
	Segmental dysfunction	5	14%	4%
	No abnormalities	21	60%	

* Arrhythmias observed during/after effort were ventricular arrhythmias in 26 patients (3 had also ST segment abnormalities), and complete atrio-ventricular block in one. Typical chest pain includes typical angina and oppressive chest pain.

Arrhythmias were observed in 34 patients: in particular, 28 patients had ventricular ectopic beats and another 6 patients experienced other kinds of arrhythmias (supraventricular tachycardia, severe bradycardia, and atrio-ventricular block).

Typical angina was complained by 32 patients, atypical precordial pain, chest discomfort, or epigastric pain (suggestive of angina equivalents) were reported by 21 patients and other atypical symptoms (dyspnea, sore throat, jaw pain, palpitation, dizziness, and syncope) were instead experienced by 11 patients. Noteworthy, 51 patients were completely asymptomatic, and the diagnosis was made based on ECG and/or echocardiographic changes. The correlations between symptoms, ECG signs of ischemia, and arrhythmias are described in Table 4.

**TABLE 4:** (A) Correlation between symptoms and ECG changes suggestive of ischemia. (B) Correlation between symptoms and ventricular arrhytmias.

(A)				
**Ischemic-like symptoms**	**ECG signs of ischemia**			
	**No**	**ST segment elevation**	**ST segment depression**	**Negative T waves**

No	8	19	14	7
Dyspnoea		2		2
Atypical chest pain		17		3
Typical angina	6	23	1	1
Atypical symptoms		4	1	2

**(B)**				

**Symptoms**	**Ventricular arrhythmias**			
	**Rare**	**Frequent**		**Ventricular tachycardia**

No	3	9		7
Dyspnoea			
Atypical chest pain	1	4		0
Typical angina	2	2		3
Atypical symptoms		9		4

An echocardiogram was performed immediately at the time of detection of cardiotoxicity in 33 patients: it showed global or segmental kinetics abnormalities in 14 patients, while it was normal in the remaining 19 patients. Troponin was dosed in 28 patients and was above the normal limits of the laboratory in 8 cases.

After the diagnosis of FP cardiotoxicity, each patient was managed on an individual basis, according to the severity of toxicity, the stage of the neoplastic disease, and the availability of alternative treatments. The patients with acute coronary syndrome or severe arrhythmias were admitted to the Intensive Care Unit (ICU) and treated according to the best clinical practice. It is important to recall that uridine triacetate, which has been proven effective for severe FP toxicity, is currently not available for use in Italy (12, 13). The patients with minor signs or symptoms were either treated in the oncologic ward under cardiologic supervision or treated on an ambulatory basis. Cardiovascular therapy was prescribed according to the type of toxicity (MI, arrhythmias, LVD) and whether it occurred at rest or it was effort-related. If severe, even life-threatening toxicities (severe MI, arrhythmias, or LVD) occurred, or if the CT was considered avoidable (i.e., adjuvant treatment in patients with mild risk of relapse) or valid alternative regimens were available, FP-based CT was interrupted. In patients with minor toxicity or a strong indication to receive FP, the treatment was continued; in other patients, instead, it was interrupted but a rechallenge was attempted months or years later because of a relapse of the disease.

Overall, FP treatment was continued or re-introduced in 35 patients (Table 5). To prevent the recurrence of a cardiotoxicity event, several strategies (alone or in combination) were used: in 7 patients capecitabine was replaced by the 5-FU infusion lasting ≤72 h; in 9 patients the FP dose was reduced by 25–50%; in 22 patients anti-ischemic and/or antiarrhythmic drugs (nitrates, calcium channel blockers, beta-blockers, ranolazine, and trimetazidine) were added to the therapy. A second rechallenge with a different approach (increase of FP dose after a successful attempt of rechallenge, or a different drug) was attempted in 7 patients. All the patients with effort-induced cardiotoxicity were screened with physical stress test during the rechallenge. The characteristics of the patients, the strategies applied and the results are reported in Table 5. Within the 35 patients who underwent the first rechallenge, 6 had again a severe cardiotoxicity event and the treatment was definitively interrupted in 5 of them (one of these patients was shifted to raltitrexed which, like FP, belongs to the CT class of anti-metabolites), instead, 4 patients had milder cardiotoxicity (evident only during stress test) and continued the therapy avoiding any physical effort and, finally, 25 patients were able to tolerate the rechallenge. Cardiotoxicity was completely prevented in 5 of the 7 patients who had an FP dose reduction only, in 10 of the 13 patients which received FP with dose reduction and anti-ischemic therapy, and in 4 of those patients who received a full dose FP and anti-ischemic therapy. Also, 4 patients with mild or no toxicity who received FP at a lower dose, experienced more severe toxicity when the drug dose was increased again. The 3 patients who shifted to raltitrexed did not have any cardiovascular adverse events and tolerated 3, 4, and 28 CT courses, respectively.

**TABLE 5:** Patients with rechallenge chemotherapy after cardiotoxicity.

PT N	Sex, age	Drug with toxicity	Type of toxicity	Rechallenge: drug	Dose	Anti-ischemic drugs	Toxicity	N of cycles
1	F, 43	5FU	Angina	5FU	75%	Diltiazem, nitrates	No	8
2	F, 47	5FU	Silent ischemia	5FU	75%		No	2
3*	M, 59	Capecitabine	Effort silent ischemia	5FU	100%	Ranolazine	Severe	1
4	F, 65	5FU	Angina	5FU	100%	Nitrates, Ranolazine, ASA	Severe	1
5*	M, 49	Capecitabine	Effort silent ischemia	Capecitabine	50%		Mild	1
6	F, 68	Capecitabine	Effort silent ischemia	Capecitabine	75%	Betablockers	Mild	1
7*	F, 51	5FU	Angina	5FU	75%		No	1
8	M, 49	5FU	Angina	5FU	100%	Nitrates	No	2
9	M, 67	Capecitabine	Effort ischemia and arrhythmias	5FU	75%	Betablockers, amlodipine	Mild	1
10*	F, 69	5FU	Atypical	5FU	75%	Diltiazem	Mild	1
11	M, 61	5FU	Effort silent ischemia	5FU	75%		No	3
12	M, 69	Capecitabine	Silent ischemia	Capecitabine	75%	Betablockers	No	2
13	M, 61	Capecitabine	Effort Arrythmias	5FU	75%		No	1
14*	F, 73	5FU	Effort ischemia and arrhythmias	5FU	60%		No	6
15	F, 61	Capecitabine	Effort angina	5FU	100%	Diltiazem, nitrates	Severe	1
16*	M, 63	5FU	Angina	5FU	75%	Nifedipine, nitrates	No	6
17	F, 42	Capecitabine	Effort angina	Capecitabine	50%	Ranolazine	No	7
18	M, 63	5FU	Angina	5FU	75%	Nifedipine, nitrates	No	5
19	M, 73	5FU	Myocardial infarction	5FU	75%	Betablockers, nitrates	No	5
20	M, 53	5FU	Angina	5FU	75%		No	3
21	F, 24	5FU	Silent ischemia	5FU	66%	Ranolazine	No	3
22	M, 58	5FU	Silent ischemia	Capecitabine	100%	Diltiazem, nitrates	No	6
23	F, 65	Capecitabine	Angina	Capecitabine	75%	Verapamil, nitrates	No	2
24	F, 43	Capecitabine	Silent ischemia, LVD	Capecitabine	66%		Severe	1
25	M, 43	Capecitabine	Effort ischemia, atypical symptoms	Capecitabine	66%	Nitrates, ASA	No	1
26	M, 42	5FU	Angina	5FU		Diltiazem, nitrates	Severe	1
27	M, 57	Capecitabine	Effort arrhythmias	5FU	100%		No	3
28	M, 46	5FU	Effort silent ischemia	5FU	75%		No	3
29	M, 68	Capecitabine	Effort ischemia, atypical symptoms	Capecitabine		Betablockers, nitrates	Severe	1
30	M, 75	Capecitabine	Effort arrhythmias	5FU	100%		No	3
31	M, 67	5FU	ischemia, atypical symptoms	5FU	100%	Nitrates	No	3
32	F, 55	5FU	Takotsubo	Raltitrexed	100%		No	28
33	M, 55	Capecitabine	Effort arrhythmias	Capecitabine	100%	Betablockers	No	3
34	M, 47	5FU	Effort angina	5FU	100%	Nitrates	No	1
35*	F, 58	Capecitabine	Effort angina	Capecitabine	75%	Trimetazidine, nitrates	No	1

				**Second rechallenge**				

	**DRUG 1^st^ rechallenge**		**Toxicity**	**Drug**	**Dose**			

3*	5FU		Severe	Raltitrexed	100%		No	4
5*	Capecitabine 50%		Mild	Capecitabine	75%		Severe	1
7*	5FU 75%		No	5FU	100%		Severe	1
10*	5FU 75%, Diltiazem		No	5FU	100%	Diltiazem	Severe	1
14*	5FU 75%		No	Capecitabine	75%		No	3
16*	5FU 75%, Nifedipine, nitrates		No	Raltitrexed	100%		No	3
35*	Capecitabine 75%, Trimetazidine, nitrates		No	Capecitabine	100%	Trimetazidine, nitrates	Mild	1

5FU, 5 Fluorouracil; ASA, Acetilsalycilc acid; LVD, left ventricular dysfunction; *, patients who had a second rechallenge with different approach.

## Discussion

In our experience, the clinical presentation of FP cardiotoxicity is extremely variable and often different from the classical description of “angina and ST-segment elevation at ECG,” which is typical of vasospastic angina.

More than one-third of our patients were completely asymptomatic and cardiotoxicity was identified on the basis of ECG changes. This prevalence of asymptomatic cases is much higher than the one reported in other retrospective studies but lower than the prevalence observed in the two prospective studies conducted in our Institution where all the patients without rest cardiotoxicity performed a stress test (14). Actually, this study includes the cases detected in the two prospective studies conducted in our Institution, in which cardiotoxicity was actively searched for with EST, and also those observed during daily clinical practice. It should be considered that, after our first observations of effort-induced cardiotoxicity and of asymptomatic ischemia in 2001 (15), we started active surveillance of cardiotoxicity even outside the prospective studies and this raised our chances of detecting the cardiotoxicity in regular clinical practice. For all patients, we perform a routine baseline ECG before the beginning of the treatment, we plan a second one after 2–4 days of CT with 5-FU or after 7–14 days of CT with capecitabine whenever possible (i.e., patients receiving in-hospital CT, patients undergoing daily radiotherapy, patients living near the hospital) and we advise the patients undergoing FP-based CT to avoid any physical effort and to promptly refer any new symptom (as chest pain, jaw pain, dyspnea, and palpitations) occurring during therapy. When a new ECG abnormality is observed and/or a new symptom is reported, the patient undergoes a cardiologic evaluation including an echocardiogram and/or stress test, if necessary, to define the diagnosis. This approach has been demonstrated to be effective in detecting several asymptomatic or oligosymptomatic toxicities, probably missed by most of the retrospective studies published so far, which included only those patients with clinical symptoms referred to the caring oncologists (16). At the same time, by advising to avoid physical efforts, the probability of eliciting effort-induced cardiotoxicity (which, according to our prospective studies with EST, accounts for half of the cases of cardiotoxicity) is reduced.

Concerning the ECG abnormalities, about half of the patients evaluated in the present study had ST-segment elevation (either alone or with specular ST segment depression), while other patients had negative T waves only or arrhythmias without typical ECG signs of ischemia. This contrasts with the hypothesis of vasospasm being the main cause of FP-related cardiotoxicity, which has been proposed for many years, and it is in support of multifactorial pathophysiology (17–21). Of note, ST-segment elevation was more frequent in the patients with rest cardiotoxicity, compared with those with stress-induced toxicity.

Other studies have reported retrospective or prospective series of FP-related cardiotoxicity, but it is not always easy to compare those data with ours, as the criteria for defining cardiotoxicity, and even the symptoms and the ECG changes, are equivocal.

In 1992, De Forni et al. prospectively studied 367 patients undergoing 96–120 h of 5-FU continuous infusion. Cardiotoxicity was observed in 28 patients (7.6%): 18 of them had angina, 12 presented cardiac collapse or pulmonary edema, and 8 patients died (5 suddenly and 3 of cardiogenic shock), ECG signs of ischemia were evident in 18 out of these 28 patients and global or segmental kinetics reduction was evident in 9 out of 16 patients who underwent an echocardiogram (22).

In a prospective study, Yilmaz et al. evaluated the role of Holter monitoring in 27 patients treated with 5FU: they did not observe any ST-T change (not even in the 2 patients who experienced chest pain); however, both a significant decrease in mean heart rate and an increase in the number of VA were reported (23).

Khan et al., in a 2012 retrospective study, reported 60 cases of “symptomatic cardiotoxicity” including 10 patients with not specified “ischemic ECG changes,” 10 with “chest pain,” 11 with ventricular tachycardia, 1 cardiac arrest, 36 with bradycardia, 18 with hypotension, 7 with hypertension, and 2 with atrio-ventricular block. The ECG repolarization abnormalities were described for 18 cases only: ST-segment elevation was detected in 5 patients, ST segment depression in 2 patients, and negative T waves in 11 patients (24).

Peng et al. (10) in 2018, published a multicentric prospective study evaluating data from 527 patients of which 161 experienced cardiotoxicity related to FP administration. In particular, 6 patients experienced a MI, 20 had heart failure (no cases of angina are reported) and 33 had “premature beats” (if ventricular or supraventricular is not specified). At ECG, a total of 105 “ischemic changes” were reported, including 70 “ST changes” and 47 “T wave changes”.

Instead, in a retrospective study by Zafar et al., only 5-FU-induced coronary vasospasm was considered and only a very low rate of cardiotoxicity was reported: although the occurrence of 5-FU-related cardiotoxicity was likely underestimated, this actually confirms our observation that typical vasospastic angina probably accounts for no more than 50% of the cases of FP cardiotoxicity (25, 26).

In a 2016 retrospective study, Dyhl-Polk et al. reported data from 452 breast cancer patients treated with capecitabine. In this study, a total of 22 cases of cardiotoxicity were diagnosed on the basis of the appearance of cardiac symptoms: chest pain in 11 patients, MI in 2 patients, arrhythmias in 5 patients (one had a cardiac arrest), and dyspnea in 3 patients (27). Two recently published studies (one retrospective and one prospective) by the same group gave results comparable to the ones obtained in our study. In the retrospective study, conducted on patients with colorectal cancer (of which 995 were treated by 5-FU and 1241 with capecitabine), 103 cases of FP-related cardiotoxicity were reported (5.2% in the 5-FU group and 4.1% in the capecitabine group). The ECG (not obtained for all patients) showed ST-segment elevation in 17 cases, ST segment depression or negative T waves in 9 and 8 cases, respectively, and VA in 6 cases. Regarding the symptoms, 45 patients had unstable angina, 23 patients experienced acute MI (10 cases with ST-segment elevation and 13 cases without ST-segment elevation), 10 patients had atypical symptoms (chest pain, dizziness, and dyspnea), 2 patients experienced a syncope secondary to atrio-ventricular or sino-atrial block and a total of 10 patients experienced sudden death or cardiac arrest (28). In the prospective study, instead, the same group of authors reported MI detected by ECG Holter in 20 patients receiving FP (18.7% of the whole group), and 16 of these patients (15% of the whole group evaluated, 80% of those with signs of ischemia) had silent ischemia (29). Six patients (5.6% of the whole study group) developed an acute coronary syndrome (in 3 cases the symptoms had been preceded by silent ischemia recorded at Holter) and 2 patients had symptomatic VT; 1 patient had a cardiac arrest after cessation of 5-FU and Holter recording revealed an ST-segment elevation. These two studies confirm some of our observations: first of all, in FP-related cardiotoxicity, ST segment depression or negative T waves are as frequent as ST-segment elevation. Secondly, that silent ischemia is rather frequent and can precede significant clinical events, such as acute coronary syndrome and/or cardiac arrest. Finally, the detection of FP-related cardiotoxicity is more than doubled in prospective studies in which planned ECG screening is performed.

It should be considered that, in our study, 24 out of 31 patients complaining of typical angina and 8 out of 9 patients with chest pain, presented ECG signs of ischemia, mostly represented by ST-segment elevation (assessed in 18 and 6 patients, respectively). This might explain why, in the studies identifying cardiotoxicity on the basis of clinical symptoms, ST-segment elevation is the most frequent ECG abnormality.

Another peculiar observation in our experience is that 55% of the patients with effort-induced cardiotoxicity had VA, which may cause syncope or sudden death. This is the main reason why we are presently giving to all the patients beginning a CT with capecitabine the advice to avoid any unusual physical effort when on therapy.

Another peculiar finding in our experience is that 60% of the echocardiograms performed shortly after detection of the cardiotoxicity were normal; it should be considered, however, that both symptoms and ECG abnormalities may vary over time, ad if the echocardiogram is not obtained during the acute episode it can be normal. Thus, a normal ECG and a normal echocardiogram in a patient who had reported angina symptoms during FP therapy but is presently asymptomatic cannot rule out the cardiotoxicity.

As regards the possibility that a dihydropyrimidine dehydrogenase (DPD) deficiency might have played a role in the cardiotoxicity of our patients, most of the cases had been observed before the routine use of the test in our Institution, and all those who were tested showed a wild-type gene. Thus, also a wild-type phenotype cannot exclude the possibility of cardiotoxicity. It should also be noted that the DPD polymorphism is a known risk factor for hematological and gastrointestinal, but not cardiac toxicity. (30, 31).

The rechallenge with the same drug after FP-induced cardiotoxicity poses a high risk of severe events and death; according to the suggestions provided by Saif et al., we limited the rechallenges to the patients with a strong indication of FP therapy and performed close monitoring with frequent ECG (or Holter monitoring), a close cardiologic follow-up and EST in selected cases (32, 33). The strategies employed to prevent cardiotoxicity when a rechallenge was considered necessary were variable and depended upon the available knowledge on FP toxicity, the anti-ischemic drugs available at different times, and also to the compliance of the patients. In the first years, we used mostly nitrates and nifedipine, according to the hypothesis of vasospasm; diltiazem was the preferred calcium channel blocker after a report on its utility in a small series of patients (34); beta-blockers were used in those patients with VA as the main manifestation of cardiotoxicity, in those with underlying coronary artery disease and in those with typical angina but with no signs of vasospasm. However, many patients were hypotensive and did not tolerate calcium channels blocker or beta blockers and others did not tolerate nitrates because of the onset of headaches. In some cases, the therapeutic approach was modified several times, using a treadmill stress test to assess the efficacy of the preventive measures, always trying to maintain the best anti-neoplastic effect, as previously described (33). Ranolazine, introduced in clinical practice in recent years, was well tolerated and it was effective in 2 patients, but not in a third. The number of patients undergoing a rechallenge is too little to allow an analysis of the efficacy of different cardiovascular treatments. However, our data suggest that the reduction of FP dose (associated with an anti-ischemic treatment if tolerated) and the shift from capecitabine to 5-FU or to a less cardiotoxic drug as raltitrexed, is probably the best approach, as already reported by other studies (35–40).

Raltitrexed is a quinazoline inhibitor of the enzyme thymidylate synthase and it is employed in the treatment of advanced malignant pleural mesothelioma [in association with cisplatin it has been demonstrated to improve the overall survival (41)] and in the treatment of advanced colorectal cancer. In patients with advanced colorectal cancer, raltitrexed has failed in demonstrating a superiority, in terms of survival outcome, when compared to 5-FU, at the cost of a higher incidence of hematological and gastrointestinal toxicity (42). However, in patients with cardiotoxicity induced by 5-FU or capecitabine, raltitrexed can represent a valid alternative, given the better cardiovascular tolerability profile (40, 42).

S1, an oral fluoropyrimidine composed of tegafur (a 5-FU prodrug), gimeracil (a dihydropyrimidine dehydrogenase, DPD, inhibitor), and potassium oxonate, is employed in Asia and in some European Countries for the treatment of different kinds of solid tumors, including advanced colorectal cancer. Lower toxicity of S-1 in the cardiovascular system could be explained by the fact that gimeracil inhibits DPD, which degrades 5-FU into its main metabolite alpha-fluoro-beta-alanine (43) (FbAL). Muneoka et al., in fact, described the case of a patient that experienced a MI after 5-FU administration and in which high levels of serum FbAL were detected. This same patient was later treated with S-1 and did not experience any additional cardiotoxicity (44).

Uracil/Tegafur (UTF), which is an oral agent composed of tegafur and uracil, is employed in Asia and also in South America in patients with advanced colorectal cancer that have experienced a cardiotoxicity event following the administration of 5-FU or capecitabine (45).

However, there is still not enough evidence regarding the potential cardiotoxicity of both S1 and UTF, thus requiring particular attention and close monitoring when employed (46).

## Conclusion

Fluoropyrimidines (FP) cardiotoxicity is an elusive clinical condition and its recognition is challenging. At least half of the patients do not complain of angina or equivalents (and dizziness should be considered a warning symptom), and the ECG abnormalities may be absent at rest ECG.

Effort-induced clinical cardiotoxicity is characterized in about 50% of the cases by VA and by atypical symptoms (including dizziness). Thus, the patients undergoing FP therapy should be discouraged from affording any unusual physical effort.

ECG ischemic changes without angina, either detected at routine ECG, at Holter or evoked by a physical effort should be not disregarded as clinically irrelevant, as they may be a sign of even severe cardiotoxicity.

Active surveillance with ECG during CT and advising the patients to refer any new symptom may increase the detection of asymptomatic or oligosymptomatic cardiotoxicity. Also, a stress test, performed during active oncologic treatment, seems necessary to rule out the occurrence of cardiotoxicity events.

## Data availability statement

The raw data supporting the conclusions of this article will be made available by the authors, without undue reservation.

## Ethics statement

This study was reviewed and approved by the Internal Review Board of the Centro di Riferimento Oncologico (CRO), National Cancer Institute. Written informed consent was not required for this study in accordance with the local legislation and institutional requirements.

## Author contributions

CL: study planning, data collection and review, writing the draft, and the final manuscript. LC and LG: analysis of the clinical data and writing the manuscript. EV: data collecting, clinical evaluation, and follow-up of the patients. EB: data analysis. MC: revision of ECG tracing and editing of the manuscript. All authors contributed to the article and approved the submitted version.

## References

[1] Ng M Cunningham D Norman AR. The frequency and pattern of cardiotoxicity observed with capecitabine used in conjunction with oxaliplatin in patients treated for advanced colorectal cancer (CRC). *Eur J Cancer.* (2005) 41:1542–6. 10.1016/j.ejca.2005.03.027 15978800

[2] Stewart T Pavlakis N Ward M. Cardiotoxicity with 5-fluorouracil and capecitabine: more than just vasospastic angina. *Intern Med J.* (2010) 40:303–7. 10.1111/j.1445-5994.2009.02144.x 20529041

[3] Kosmas C Kallistratos MS Kopterides P Syrios J Skopelitis H Mylonakis N Cardiotoxicity of fluoropyrimidines in different schedules of administration: a prospective study. *J Cancer Res Clin Oncol.* (2008) 134:75–82. 10.1007/s00432-007-0250-9 17636329 PMC12161746

[4] McAndrew EN Jassal DS Goldenberg BA Kim CA. Capecitabine-mediated heart failure in colorectal cancer: a case series. *Eur Heart J Case Rep.* (2021) 5:ytab079. 10.1093/ehjcr/ytab079 33709051 PMC7936921

[5] Layoun ME Wickramasinghe CD Peralta MV Yang EH. Fluoropyrimidine-induced cardiotoxicity: manifestations, mechanisms, and management. *Curr Oncol Rep.* (2016) 18:35. 10.1007/s11912-016-0521-1 27113369

[6] Kanduri J More LA Godishala A Asnani A. Fluoropyrimidine-associated cardiotoxicity. *Cardiol Clin.* (2019) 37:399–405. 10.1016/j.ccl.2019.07.004 31587781

[7] More LA Lane S Asnani A. 5-FU cardiotoxicity: vasospasm, myocarditis, and sudden death. *Curr Cardiol Rep.* (2021) 23:17. 10.1007/s11886-021-01441-2 33537861

[8] Lestuzzi C Viel E Picano E Meneguzzo N. Coronary vasospasm as a cause of effort-related myocardial ischemia during low-dose chronic continuous infusion of 5-fluorouracil. *Am J Med.* (2001) 111:316–8. 10.1016/s0002-9343(01)00808-7 11566462

[9] Lestuzzi C Vaccher E Talamini R Lleshi A Meneguzzo N Viel E Effort myocardial ischemia during chemotherapy with 5-Fluorouracil: an underestimated risk. *Ann Oncol.* (2014) 25:1059–64. 10.1093/annonc/mdu055 24558023

[10] Peng J Dong C Wang C Li W Yu H Zhang M Cardiotoxicity of 5-fluorouracil and capecitabine in Chinese patients: a prospective study. *Cancer Commun.* (2018) 38:22. 10.1186/s40880-018-0292-1 PMC5953402 29764506

[11] Sara JD Kaur J Khodadadi R Rehman M Lobo R Chakrabarti S 5-fluorouracil and cardiotoxicity: a review. *Ther Adv Med Oncol.* (2018) 10:1758835918780140. 10.1177/1758835918780140 29977352 PMC6024329

[12] Ma WW Saif MW El-Rayes BF Fakih MG Cartwright TH Posey JA Emergency use of uridine triacetate for the prevention and treatment of life-threatening 5-fluorouracil and capecitabine toxicity. *Cancer.* (2017) 123:345–56. 10.1002/cncr.30321 27622829 PMC5248610

[13] Raber I Frazer MB Zerillo JA Asnani A. Uridine triacetate for severe fluoropyrimidine cardiotoxicity in a patient with thymidylate synthase gene variants. *JACC CardioOncol.* (2020) 2:329–32. 10.1016/j.jaccao.2020.04.005 34396241 PMC8352086

[14] Lestuzzi C Stolfo D De Paoli A Banzato A Buonadonna A Bidoli E Cardiotoxicity from capecitabine chemotherapy: prospective study of incidence at rest and during physical exercise. *Oncologist.* (2022) 27:e158–67. 10.1093/oncolo/oyab035 35641220 PMC8895550

[15] Hrovatin E Viel E Lestuzzi C Tartuferi L Zardo F Brieda M Severe ventricular dysrhythmias and silent ischemia during infusion of the antimetabolite 5-fluorouracil and cis-platin. *J Cardiovasc Med.* (2006) 7:637–40. 10.2459/01.JCM.0000237914.12915.dd 16858245

[16] Jensen SA Sørensen JB. Risk factors and prevention of cardiotoxicity induced by 5-fluorouracil or capecitabine. *Cancer Chemother Pharmacol.* (2006) 58:487–93. 10.1007/s00280-005-0178-1 16418875

[17] Mosseri M Fingert HJ Varticovski L Chokshi S Isner JM. In vitro evidence that myocardial ischemia resulting from 5-fluorouracil chemotherapy is due to protein kinase C-mediated vasoconstriction of vascular smooth muscle. *Cancer Res.* (1993) 53:3028–33. 8391384

[18] Salepci T Seker M Uyarel H Gumus M Bilici A Ustaalioglu BB 5-Fluorouracil induces arterial vasoconstrictions but does not increase angiotensin II levels. *Med Oncol.* (2010) 27:416–20. 10.1007/s12032-009-9226-8 19415535

[19] Sudhoff T Enderle MD Pahlke M Petz C Teschendorf C Graeven U 5-Fluorouracil induces arterial vasocontractions. *Ann Oncol.* (2004) 15:661–4. 10.1093/annonc/mdh150 15033676

[20] Polk A Vistisen K Vaage-Nilsen M Nielsen DL. A systematic review of the pathophysiology of 5-fluorouracil-induced cardiotoxicity. *BMC Pharmacol Toxicol.* (2014) 15:47. 10.1186/2050-6511-15-47 25186061 PMC4170068

[21] Focaccetti C Bruno A Magnani E Bartolini D Principi E Dallaglio K Effects of 5-fluorouracil on morphology, cell cycle, proliferation, apoptosis, autophagy and ROS production in endothelial cells and cardiomyocytes. *PLoS One.* (2015) 10:e0115686. 10.1371/journal.pone.0115686 25671635 PMC4324934

[22] de Forni M Malet-Martino MC Jaillais P Shubinski RE Bachaud JM Lemaire L Cardiotoxicity of high-dose continuous infusion fluorouracil: a prospective clinical study. *J Clin Oncol.* (1992) 10:1795–801. 10.1200/JCO.1992.10.11.1795 1403060

[23] Yilmaz U Oztop I Ciloglu A Okan T Tekin U Yaren A 5-fluorouracil increases the number and complexity of premature complexes in the heart: a prospective study using ambulatory ECG monitoring. *Int J Clin Pract.* (2007) 61:795–801. 10.1111/j.1742-1241.2007.01323.x 17493091

[24] Khan MA Masood N Husain N Ahmad B Aziz T Naeem A. A retrospective study of cardiotoxicities induced by 5-fluouracil (5-FU) and 5-FU based chemotherapy regimens in Pakistani adult cancer patients at Shaukat Khanum Memorial Cancer Hospital & Research Center. *J Pak Med Assoc.* (2012) 62:430–4. 22755303

[25] Zafar A Drobni ZD Mosarla R Alvi RM Lei M Lou UY The incidence, risk factors, and outcomes with 5-fluorouracil-associated coronary vasospasm. *JACC CardioOncol.* (2021) 3:101–9. 10.1016/j.jaccao.2020.12.005 33817666 PMC8018593

[26] Dyhl-Polk A Vaage-Nilsen M Schou M Vistisen KK Lund CM Kümler T Incidence and risk markers of 5-fluorouracil and capecitabine cardiotoxicity in patients with colorectal cancer. *Acta Oncol.* (2020) 59:475–83. 10.1080/0284186X.2019.1711164 31931649

[27] Polk A Shahmarvand N Vistisen K Vaage-Nilsen M Larsen FO Schou M Incidence and risk factors for capecitabine-induced symptomatic cardiotoxicity: a retrospective study of 452 consecutive patients with metastatic breast cancer. *BMJ Open.* (2016) 6:e012798. 10.1136/bmjopen-2016-012798 PMC5073470 27798021

[28] Dyhl-Polk A Schou M Vistisen KK Sillesen AS Serup-Hansen E Faber J Myocardial ischemia induced by 5-fluorouracil: a prospective electrocardiographic and cardiac biomarker study. *Oncologist.* (2021) 26:e403–13. 10.1002/onco.13536 32959474 PMC7930422

[29] Becker K Erckenbrecht JF Häussinger D Frieling T. Cardiotoxicity of the antiproliferative compound fluorouracil. *Drugs.* (1999) 57:475–84. 10.2165/00003495-199957040-00003 10235688

[30] Kim W Cho YA Kim DC Lee KE. Elevated risk of fluoropyrimidine-associated toxicity in European patients with *DPYD* genetic polymorphism: a systematic review and meta-analysis. *J Pers Med.* (2022) 12:225. 10.3390/jpm12020225 35207713 PMC8875904

[31] Depetris I Marino D Bonzano A Cagnazzo C Filippi R Aglietta M Fluoropyrimidine-induced cardiotoxicity. *Crit Rev Oncol Hematol.* (2018) 124:1–10. 10.1016/j.critrevonc.2018.02.002 29548480

[32] Wasif Saif M Shah MM Shah AR. Fluoropyrimidine-associated cardiotoxicity: revisited. *Expert Opin Drug Saf.* (2009) 8:191–202. 10.1517/14740330902733961 19309247

[33] Lestuzzi C Crivellari D Rigo F Viel E Meneguzzo N. Capecitabine cardiac toxicity presenting as effort angina: a case report. *J Cardiovasc Med.* (2010) 11:700–3. 10.2459/JCM.0b013e328332e873 20093950

[34] Ambrosy AP Kunz PL Fisher GA Witteles RM. Capecitabine-induced chest pain relieved by diltiazem. *Am J Cardiol.* (2012) 110:1623–6. 10.1016/j.amjcard.2012.07.026 22939579

[35] Saif MW Garcon MC Rodriguez G Rodriguez T. Bolus 5-fluorouracil as an alternative in patients with cardiotoxicity associated with infusion 5-fluorouracil and capecitabine: a case series. *In Vivo.* (2013) 27:531–4. 23812226

[36] Deboever G Hiltrop N Cool M Lambrecht G. Alternative treatment options in colorectal cancer patients with 5-fluorouracil- or capecitabine-induced cardiotoxicity. *Clin Colorectal Cancer.* (2013) 12:8–14. 10.1016/j.clcc.2012.09.003 23102544

[37] Kelly C Bhuva N Harrison M Buckley A Saunders M. Use of raltitrexed as an alternative to 5-fluorouracil and capecitabine in cancer patients with cardiac history. *Eur J Cancer.* (2013) 49:2303–10. 10.1016/j.ejca.2013.03.004 23583220

[38] Ransom D Wilson K Fournier M Simes RJ Gebski V Yip D Final results of Australasian Gastrointestinal Trials Group ARCTIC study: an audit of raltitrexed for patients with cardiac toxicity induced by fluoropyrimidines. *Ann Oncol.* (2014) 25:117–21. 10.1093/annonc/mdt479 24299960

[39] Khan K Rane JK Cunningham D Rao S Watkins D Starling N Efficacy and cardiotoxic safety profile of raltitrexed in fluoropyrimidines-pretreated or high-risk cardiac patients with GI malignancies: large single-center experience. *Clin Colorectal Cancer.* (2019) 18:64–71.e1. 10.1016/j.clcc.2018.09.010 30404764

[40] Sorrentino MF Kim J Foderaro AE Truesdell AG. 5-fluorouracil induced cardiotoxicity: review of the literature. *Cardiol J.* (2012) 19:453–8. 10.5603/cj.2012.0084 23042307

[41] van Meerbeeck JP Gaafar R Manegold C Van Klaveren RJ Van Marck EA Vincent M Randomized phase III study of cisplatin with or without raltitrexed in patients with malignant pleural mesothelioma: an intergroup study of the European organisation for research and treatment of cancer lung cancer group and the National cancer institute of Canada. *J Clin Oncol.* (2005) 23:6881–9. 10.1200/JCO.20005.14.589 16192580

[42] Maughan TS James RD Kerr DJ Ledermann JA McArdle C Seymour MT Comparison of survival, palliation, and quality of life with three chemotherapy regimens in metastatic colorectal cancer: a multicentre randomised trial. *Lancet.* (2002) 359:1555–63. 10.1016/s0140-6736(02)08514-8 12047964

[43] Yamada Y Hamaguchi T Goto M Muro K Matsumura Y Shimada Y Plasma concentrations of 5-fluorouracil and F-beta-alanine following oral administration of S-1, a dihydropyrimidine dehydrogenase inhibitory fluoropyrimidine, as compared with protracted venous infusion of 5-fluorouracil. *Br J Cancer.* (2003) 89:816–20. 10.1038/sj.bjc.6601224 12942110 PMC2394492

[44] Muneoka K Shirai Y Yokoyama N Wakai T Hatakeyama K. 5-Fluorouracil cardiotoxicity induced by alpha-fluoro-beta-alanine. *Int J Clin Oncol.* (2005) 10:441–3. 10.1007/s10147-005-0516-7 16369751

[45] Shiga T Hiraide M. Cardiotoxicities of 5-fluorouracil and other fluoropyrimidines. *Curr Treat Options Oncol.* (2020) 21:27. 10.1007/s11864-020-0719-1 32266582 PMC7138764

[46] Osterlund P Kinos S Pfeiffer P Salminen T Kwakman JJM Frödin JE Continuation of fluoropyrimidine treatment with S-1 after cardiotoxicity on capecitabine- or 5-fluorouracil-based therapy in patients with solid tumours: a multicentre retrospective observational cohort study. *ESMO Open.* (2022) 7:100427. 10.1016/j.esmoop.2022.100427 35798468 PMC9291631

